# Simulating Habitat Suitability Changes of Threadfin Porgy (*Evynnis cardinalis*) in the Northern South China Sea Using Ensemble Models Under Medium-to-Long-Term Future Climate Scenarios

**DOI:** 10.3390/biology14030236

**Published:** 2025-02-26

**Authors:** Junyi Zhang, Jiajun Li, Yancong Cai, Kui Zhang, Youwei Xu, Zuozhi Chen, Shannan Xu

**Affiliations:** 1 South China Sea Fisheries Research Institute, Chinese Academy of Fishery Sciences, Guangzhou 510300, China; zjy20241029@163.com (J.Z.); onion-20062006@163.com (Y.C.); zhangkui@scsfri.ac.cn (K.Z.); xuyouwei@scsfri.ac.cn (Y.X.); chenzuozhi@scsfri.ac.cn (Z.C.); 2 Key Laboratory for Sustainable Utilization of Open-Sea Fishery, Ministry of Agriculture and Rural Affairs, Guangzhou 510300, China

**Keywords:** *Evynnis cardinalis*, ensemble model, climatic change, Biomod2, northern South China Sea

## Abstract

The impact of global warming on fish distribution is a key factor in fishery management and sustainable development. However, limited knowledge exists regarding the influence of environmental factors on the distribution of *Evynnis cardinalis* under climate change. This study addresses this gap by predicting the species distribution under current conditions and three future climate scenarios (SSP126, SSP370, and SSP585) using five individual models and four ensemble models. The results demonstrate that the ensemble models outperform the single models, with majority voting (EMca) achieving the highest accuracy (ROC = 0.97, TSS = 0.85). Bathymetry (BM) and the sea surface height (SSH) are the primary factors influencing the distribution. The predictions indicate that the currently suitable habitats of *E. cardinalis* are primarily located in the Beibu Gulf region of the northern South China Sea. Under future climate scenarios, suitable habitat areas are expected to expand to higher latitudes and deeper waters, though highly suitable habitats in the western Guangdong coastal waters, western Beibu Gulf, and southwestern offshore waters of Hainan Island will significantly decrease.

## Simple Summary

This study examines the impact of climate change on the distribution of threadfin porgy in the northern South China Sea. We simulated the species’ distribution under both current and future climate conditions. The results show that water depth and sea surface height are the primary factors influencing its distribution, with the highest probability of activity at a depth of 44 m. Under future climate scenarios, suitable habitats are projected to expand, moving toward higher latitudes and deeper waters. However, the area of highly suitable habitats is expected to decrease significantly.

## 1. Introduction

Climate change (CC) is primarily driven by human activities and is occurring at an unprecedented rate [1]. It has significantly impacted marine environmental conditions, leading to phenomena such as polar glacier melting, sea level rise, and ocean acidification [2,3,4,5]. In marine ecosystems, the distribution and abundance of marine organisms are closely linked to environmental factors such as chlorophyll concentration, seawater temperature, salinity, and dissolved oxygen [6,7,8,9]. The strong mobility of fish enables them to locate suitable environments for reproduction [10]. At present, many fish species are migrating towards the poles at rates ranging from tens to hundreds of kilometers per decade [11]. This shift has resulted in biological invasions in high-latitude regions and species extinctions in low-latitude regions [12]. Rising ocean temperatures have caused larger fish species to shrink in size and experience significant declines in abundance [13]. Thus, understanding the current and future distribution changes of *Evynnis cardinalis* is essential for promoting sustainable fishery development and implementing effective marine management strategies [14].

**Figure 1:**
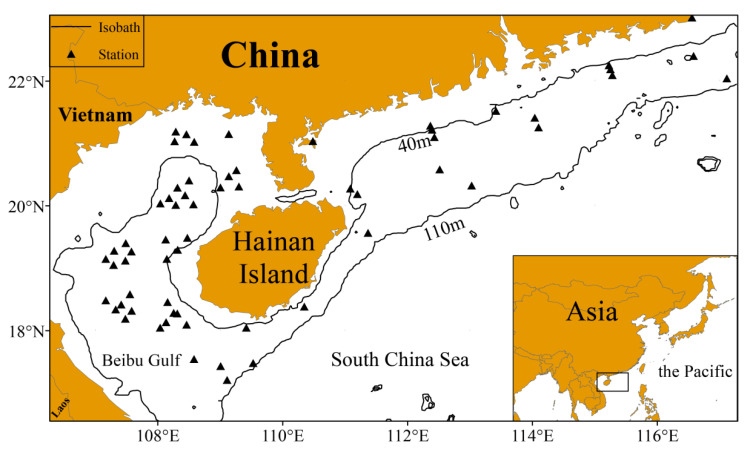
The fishery stations that were surveyed at least once from the summer of 2014 to the spring of 2017. The solid black lines represent the 40 and 110 m isobaths, and the triangles represent the sampling stations.

The South China Sea (SCS) is a marginal sea located in the tropical and subtropical waters of southeast Asia (Figure 1) [15]. It is rich in fishery resources, serving as an important marine fishing ground, with nearly 1500 fish species inhabiting its waters [16,17]. The main commercial fish species are concentrated in the northern South China Sea (NSCS) (water depth < 200 m) [18]. Currently, most fish species are experiencing overfishing [19]. In particular, the abundance of demersal fish species has significantly decreased [20], and their proportion in the total catch has declined, indicating that they are no longer the dominant species [21]. Studies indicate that demersal fish are a vital component of the marine ecosystem. Their high ecological niche allows them to play a key role in regulating populations of organisms at lower trophic levels [22]. However, there remains a lack of scientific evidence on the extent to which climate change affects the future spatial distribution of demersal fish in the NSCS. Thus, investigating the relationship between demersal fish in the NSCS and environmental factors, as well as predicting the impacts of climate change on their distribution, is essential for restoring demersal fish populations and ensuring the sustainable development of fisheries.

The threadfin porgy (*E. cardinalis*) serves as an ideal species for examining the impact of climate change on the distribution of economically important fish in the NSCS. As a nearshore warm-water demersal fish, *E. cardinalis* holds significant economic value [23]. It is widely distributed across the waters of China, Indonesia, Japan, and other western Pacific countries. In China, its primary distribution includes the East China Sea and the NSCS [24]. In the NSCS, the Beibu Gulf is the main production area of *E. cardinalis*, where it represents a key target for bottom trawl fisheries [25]. Compared with the 1990s, its resources showed some recovery in the early 21st century. However, it continues to face overfishing pressures, particularly due to the excessive harvest of juvenile fish [26]. Studies indicate that *E. cardinalis* occupies an increasingly significant ecological niche [27], playing a critical role in maintaining the flow of matter and energy within the food chain [28]. Despite this, recent years have witnessed a renewed decline in its population [29]. Understanding the relationship between *E. cardinalis* and its environmental factors is essential for effective conservation efforts and the sustainable utilization of fishery resources.

Species distribution models (SDMs) are widely used to study the relationship between species and their environment [30,31,32,33]. In fisheries, SDMs are commonly used for applications such as predicting the distribution of fished species under current and future climate conditions [34,35]. Various types of SDMs exist, including generalized additive models (GAMs), maximum entropy networks (MAXNETs), and generalized linear models (GLMs), among others [36,37,38]. However, differences in computational methods, as well as variations in species and dataset inputs, often result in inconsistent outputs across SDMs [39]. Consequently, selecting the most suitable model for predicting species distributions remains a challenge [40]. *Biomod2* (version 4.2-4) provides tools for constructing ensemble models, such as majority voting (EMca), weighted averaging (EMwmean), simple averaging (EMmean), and median-based models (EMmedian). These ensemble models combine predictions from multiple single models, thereby reducing uncertainties and errors in both data and model outputs [41].

This study employs five individual SDMs from *Biomod2*, alongside an ensemble model constructed from these individual models, to investigate the current distribution range and habitat suitability changes of *E. cardinalis* in the NSCS under three future climate scenarios (SSP126, SSP370, and SSP585). The main objective of this research is to accurately simulate the current and future potential distribution range by identifying the best model, reveal the key environmental factors influencing its distribution, and predict the distribution changes in the 2050s and 2090s under different climate scenarios. The findings provide a scientific basis for the management and strategic planning of *E. cardinalis* fisheries, aiming to support the optimization of fishing activities and ensuring the sustainable utilization of this species’ resources.

## 2. Materials and Methods

### 2.1. Data Sources

#### 2.1.1. Occurrence Data of *E. cardinalis*

This study utilized data from fishing logs collected by scientific research vessels in the NSCS from the summer of 2014 to the spring of 2017. The dataset included latitude and longitude information of fishing locations, as well as fish species, body length, and weight measurements. All fish were caught by bottom trawling for one hour. On the deck, they were sorted, and their quantities, body lengths, and weights were measured. On average, three fishing stations were surveyed per day. Each season, a voyage lasting approximately 1–2 months was carried out. In this study, we only considered whether *E. cardinalis* was present at the surveyed stations. To enhance data stability and ensure accuracy and reliability, only locations that appeared four or more times across eight independent surveys were included as species occurrence data. Sites with zero catches were excluded from the analysis. The repeated occurrence of these selected locations underscores their representativeness in defining the living environment of *E. cardinalis*. This provided reliable data for understanding the ecological habits and distribution range of the species. Ultimately, 60 species occurrence points were selected as input data for the model to predict the distribution of *E. cardinalis*.

#### 2.1.2. Environmental Data Sources

**Figure 2:**
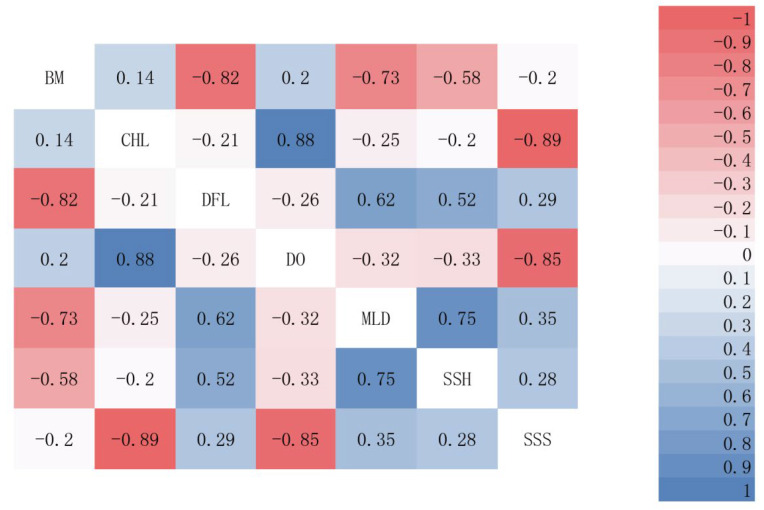
The Pearson correlation heatmap of seven environmental factors. The absolute value of the Pearson correlation coefficient ranges from 0 to 1, and the higher the absolute value, the higher the degree of correlation.

Sea bottom temperature (SBT), bathymetry (BM), and sea surface salinity (SSS) are key environmental factors influencing the distribution of *E. cardinalis*, a demersal fish [26]. Additionally, other factors, including chlorophyll (CHL), distance from land (DFL), dissolved oxygen (DO), mixed layer depth (MLD), and sea surface height above geoid (SSH), also play significant roles in shaping the habitat preferences of demersal fish [31,42]. Based on these findings, SBT, BM, SSS, CHL, DFL, DO, MLD, and SSH were selected as the predictor variables for modeling the distribution of *E. cardinalis* (Figure 2).

In this study, CHL and DO data were sourced from the Global Ocean Biogeochemistry Hindcast provided by the Copernicus Marine Environment Monitoring Service (CMEMS) (http://marine.copernicus.eu, accessed on 2 June 2024) at a resolution of 0.25° × 0.25°. MLD, SSS, SBT, and SSH data were obtained from the Global Ocean Physics Reanalysis of CMEMS with a resolution of 1/12° × 1/12°. Additionally, DFL and BM data were sourced from Global Fishing Watch (https://globalfishingwatch.org, accessed on 5 June 2024) with resolutions of 0.01° × 0.01° and 1/120° × 1/120°, respectively. To analyze distribution changes over time, the medium-term (2050s: average environmental data from 2050 to 2059) and long-term (2090s: average environmental data from 2090 to 2099) periods were set. Data under three Shared Socioeconomic Pathway (SSP) scenarios were collected from the Bio-ORACLE data center (https://www.bio-oracle.org/downloads-to-email.php, accessed on 26 May 2024): SSP126 (low greenhouse gas emissions), SSP370 (medium greenhouse gas emissions), and SSP585 (high greenhouse gas emissions). ArcGIS 10.8 was used to standardize the spatial resolution of both current and future environmental variables to 0.05° × 0.05° and crop them to the same study area (16.5° N–23° N, 106.3° E–117.3° E).

Ensuring weak correlations among selected environmental variables was crucial when constructing SDMs. First, the raster calculator tool in ArcGIS 10.8 was used to calculate the average values of eight environmental variables from 2014 to 2017. The correlations among these variables were then analyzed (Figure 2). A threshold of r = |0.7| was applied to minimize the impact of multicollinearity on the model [43]. The analysis revealed that BM was negatively correlated with DFL and MLD but positively correlated with SBT. Similarly, SSS was negatively correlated with CHL and DO. Based on these correlations, BM was selected as a research variable, as it better explains the distribution patterns of *E. cardinalis* compared to temperature, which primarily influences the growth and metabolism of marine organisms [44]. For SSS, it was chosen over DO and CHL because changes in SSS affect nutrient concentrations [45], whereas CHL indirectly influences oxygen levels through photosynthesis. Finally, BM, SSH, and SSS were selected as the key environmental variables for modeling the distribution of *E. cardinalis.*

### 2.2. Model Construction and Simulation of Potential Habitats of E. cardinalis

#### 2.2.1. Selection and Construction of Single Models

In this research, all species distribution models (SDMs) were implemented through the utilization of Biomod2 (https://cran.r-project.org/web/packages/biomod2/index.html, accessed on 2 May 2024) within R (https://www.R-project.org/, accessed on 27 May 2024). Biomod2 encompasses five individual single-model algorithms that were incorporated into the model construction: the generalized linear model (GLM), the generalized additive model (GAM), flexible discriminant analysis (FDA), multivariate adaptive regression splines (MARS), and the maximum entropy network (MAXNET). Among these, MAXNET stands distinct as it does not necessitate pseudo-absence data. In contrast, the remaining four algorithms, namely, GLM, GAM, FDA, and MARS, rely on both presence and pseudo-absence data to augment the precision and stability of species distribution forecasts [46]. To fulfill this prerequisite, an equivalent quantity of pseudo-absence data points was randomly generated. The modeling procedure adhered to the default configurations stipulated by Biomod2. Each single-model algorithm was executed ten times to guarantee a comprehensive and reliable evaluation. The dataset was partitioned into a training subset (constituting 70%) and a validation subset (amounting to 30%) for the assessment of model efficacy [47,48]. Overall, five single models were constructed, with each model undergoing ten iterations, culminating in a total of 50 models being employed to simulate the extant habitat distribution of *E. cardinalis*.

#### 2.2.2. Selection and Construction of Ensemble Models

A single species distribution model (SDM) inherently has uncertainties and algorithmic variations, which can reduce prediction accuracy [49]. To address these limitations, integrated species distribution models (ensemble models) combine predictions from multiple individual SDMs using various aggregation methods, thereby reducing model uncertainty [50]. By integrating multiple models, the ensemble approach effectively captures the true “signal” of a species’ ecological niche while mitigating the “noise” introduced by data and structural uncertainties [51]. This method provides broader input data and demonstrates significant advantages over single SDMs, resulting in improved accuracy and enhanced generalizability [52]. In this study, an ensemble model was constructed by combining predictions from five individual models using the default aggregation methods in the biomod2 package, including weighted average, simple average, median, and majority voting. The ensemble model was further used to improve prediction accuracy for the potential habitats of marine species, a method widely applied in similar research [53]. Additionally, the “bm_PlotResponseCurves” function in the *biomod2* package was utilized to generate response curves, visually analyzing the relationships between environmental variables and the occurrence probability of *E. cardinalis*. This analysis provided insights into how environmental factors influence the species’ distribution and helped identify optimal habitat conditions. Finally, the current distribution map of *E. cardinalis* was established, and its potential distribution regions for the 2050s and 2090s were predicted under three future climate scenarios.

#### 2.2.3. Evaluation of the Accuracy of Single and Ensemble Models

Model evaluation metrics play a significant role in pinpointing the most suitable model and augmenting the precision of predictions [54]. Among the array of available metrics, the receiver operating characteristic (ROC) and the true skill statistic (TSS) are prevalently adopted and acknowledged as fundamental evaluation benchmarks within the domain of SDMs [55,56]. The ROC curve demonstrates the consequences of diverse decision thresholds by contemplating all conceivable permutations of accurate and inaccurate decisions, thus endowing a thoroughgoing appraisal of model performance [57]. In contrast, the TSS amalgamates sensitivity (true positive rate) and specificity (true negative rate) into a unified metric, proffering a well-rounded evaluation of the model’s proficiency in accurately forecasting both the occurrence and non-occurrence of the subject matter [40]. Generally, the thresholds associated with these indicators are routinely employed to appraise model performance. To be more specific, an ROC value exceeding 0.7 signifies a favorable prediction aptitude, whilst a TSS value under 0.4 implies a deficient predictive precision [58].

#### 2.2.4. Alterations in the Habitat Within Future Climate Scenarios for the Projection of *E. cardinalis*

Within the framework of SDMs, the habitat suitability index (HSI) serves as an indicator of the occurrence probability of a species. The values of HSI span from 0 (signifying no occurrence) to 1 (denoting certain occurrence). For each individual SDM, the HSI was computed by taking the average of the outcomes obtained from 10 iterations. The HSI of the ensemble model was obtained by integrating the prediction outcomes of individual SDMs through methods like EMwmean, EMmean, EMca, and EMmedian [42].

To evaluate the changes in the distribution range of *E. cardinalis* under future climate conditions, a quantitative analysis of its habitat area was performed. Using ArcGIS 10.8, distribution maps of *E. cardinalis* habitats were created, and the potential habitat areas under three different climate scenarios were quantified. The habitat suitability index (HSI) was classified into four categories: unsuitable area (0 < HSI ≤ 0.25), low suitability area (0.25 < HSI ≤ 0.5), moderate suitability area (0.5 < HSI ≤ 0.75), and highly suitable area (0.75 < HSI ≤ 1). These classified areas were then compared with the current HSI-based habitat areas to assess the impact of climate change on the distribution of *E. cardinalis*.

## 3. Results

### 3.1. Evaluation of Single and Ensemble Models Performance

#### 3.1.1. Assessment of the Performance of Single Models

Based on the outcomes of the individual models (Table 1), MARS and FDA emerged as the top-performing models in terms of accuracy among the five models under consideration. Specifically, MARS attained an ROC value of 0.967 and a TSS value of 0.846. Similarly, FDA recorded an ROC value of 0.957 and a TSS value of 0.846. Conversely, GLM and MAXNET demonstrated a relatively inferior performance. GLM had an ROC value of 0.898 and a TSS value of 0.720, while MAXNET achieved an ROC value of 0.942 and a TSS value of 0.783. Notably, all of the single models achieved TSS values > 0.7 and ROC values > 0.8, indicating the high predictive accuracy and overall reliability of the single-model predictions.

#### 3.1.2. Evaluation of the Significance of Environmental Factors

Among the four ensemble models generated using different algorithms (Table 2), EMca demonstrated the best performance, achieving an ROC of 0.97 and a TSS value of 0.85. In contrast, EMmedian showed the lowest performance, with an ROC of 0.961 and a TSS value of 0.833. EMca, as a simple yet highly effective method, significantly improves the model accuracy and stability [59]. It exhibits a remarkable performance within the context of limited computing resources [60]. Furthermore, it is endowed with the capacity to engender more optimal parameter combinations through the multi-parent recombination paradigm and can precisely determine the weight combinations that are most propitious for forecasting the distribution of specific species [61]. Therefore, we selected EMca as the optimal ensemble model for simulating the current distribution of *E. cardinalis* and applied it in the final predictions.

### 3.2. Contributions of Environmental Variables to Models and Response Curves

**Figure 3:**
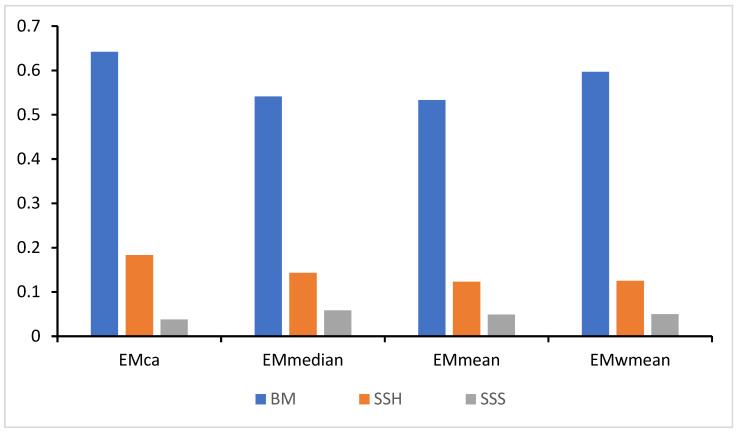
Contribution rate of environmental factors within ensemble models. The meanings represented by the abbreviated letters: majority voting (EMca), weighted averaging (EMwmean), simple averaging (EMmean), median-based models (EMmedian), bathymetry (BM), sea surface salinity (SSS), and sea surface height above geoid (SSH).

BM and SSH were determined to be the principal factors that have an impact on the distribution of *E. cardinalis*. Through the calculation of the average contribution rates of environmental factors within the four ensemble models, the outcomes (Figure 3) demonstrate that BM made a contribution ranging from 53.3% to 64.2%, and SSH contributed between 12.3% and 18.3%. Collectively, these two factors accounted for more than 60% of the total contribution, thereby verifying their dominant position in influencing the distribution pattern of *E. cardinalis*. In contrast, the average contribution rate of SSS was found to be 4.9%, which indicates that it exerts a secondary yet significant influence on the distribution of the species.

**Figure 4:**
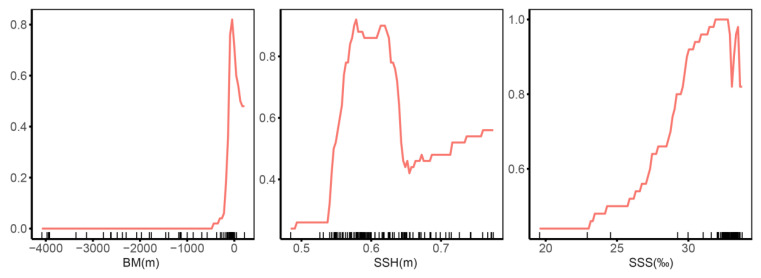
Response curves of environmental factors pertaining to the top-performing ensemble model. The red line represents the changes in the occurrence probability of *E. cardinalis* in different environments.

Environmental response curves offer significant and valuable perspectives on the connection between the probability of species occurrence and the conditions of suitable habitats (Figure 4). Among the three environmental factors in question, both BM and SSH display more conspicuous variations in their response curves, which indicates their relatively higher contribution rates and sensitivities. In the case of BM, when its value reaches 44 m, the occurrence probability of *E. cardinalis* attains its peak at 0.84. As for SSH, the first peak in the occurrence probability emerges at 0.57 m, and a second peak can be noticed at 0.61 m, where the probability stabilizes at around 0.90. The response curve for SSS showcases two separate peaks, specifically at 32.5‰ and 33.3‰, with the corresponding occurrence probabilities being 0.986 and 0.816, respectively. This implies that SSS also has an impact on influencing the habitat suitability; however, its overall sensitivity is lower when compared to that of BM and SSH.

### 3.3. Distribution of the Current Habitat and Alterations in Future Habitat for E. cardinalis

**Figure 5:**
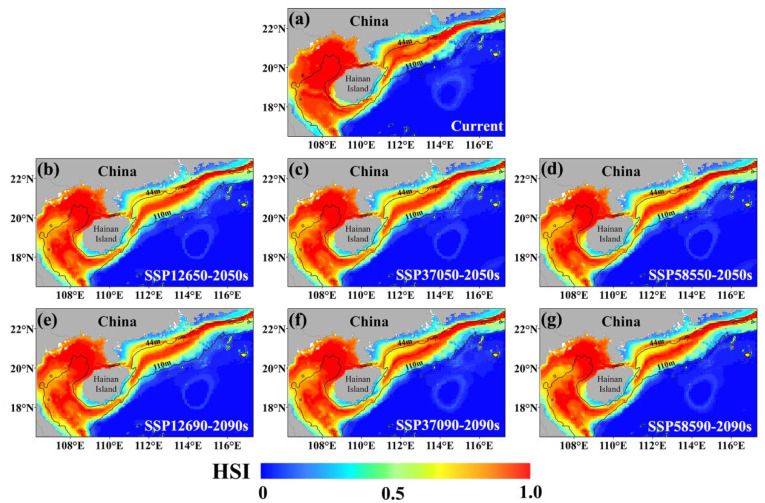
Using EMca to simulate the distribution of suitable habitats for *E. cardinalis* in the NSCS under current (**a**) and three future climate scenarios of SSP126 (**b**,**e**), SSP370 (**c**,**f**), and SSP585 (**d**,**g**) in the medium and long term. HSI represents the habitat suitability index, ranging from 0 to 1.

The ensemble model was employed to simulate the probability distribution of *E. cardinalis* in the NSCS under the existing climate conditions and the prospective scenarios of SSP126, SSP370, and SSP585 (Figure 5). Presently, *E. cardinalis* predominantly occupies the waters whose depths are no more than 110 m in the NSCS. The areas that are suitable for its habitation are mainly found in the Beibu Gulf, and the highly suitable zones are chiefly concentrated within this gulf area. Under the three future climate scenarios, a general tendency of contraction can be seen in the highly suitable habitat areas of *E. cardinalis*. Remarkably, when contrasted with the current scope of highly suitable habitats, considerable decreases are noticed in the 2050s under the SSP126 and SSP370 scenarios. By contrast, the reduction in the highly suitable habitat area is less evident under the SSP585 scenario (Table 3).

**Figure 6:**
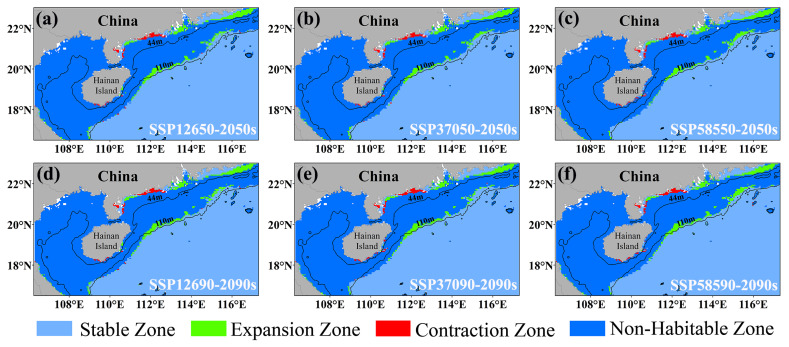
The mid- and long-term distribution of *E. cardinalis* was predicted under the climate scenarios of SSP126 (**a**,**d**), SSP370 (**b**,**e**), and SSP585 (**c**,**f**). The stable area refers to the region where the HSI is greater than or equal to 0.25 under both current and future climate scenarios. The expansion area refers to the region where the current HSI is less than 0.25 but becomes greater than or equal to 0.25 under future climate scenarios. The contraction area refers to the region where the current HSI is greater than or equal to 0.25 but becomes less than 0.25 under future climate scenarios. The unsuitable area refers to the region where the HSI is less than 0.25 under both current and future climate scenarios.

To better understand the changes in the distribution of suitable habitats for *E. cardinalis* under future climate conditions, the current distribution was used as a reference to compare changes in suitable habitats (HSI ≥ 0.25) and unsuitable habitats (HSI < 0.25) in the 2050s and 2090s. The results (Figure 6) show that under various climate scenarios, *E. cardinalis* exhibits a shrinking trend near the Guangdong coast, while expanding into higher and lower latitudes and deeper waters. This expansion trend becomes more pronounced as climate change intensifies and time progresses. The quantitative analysis of the suitable habitat areas indicates that the rate of expansion exceeds the rate of contraction. Under the three climate scenarios, the total suitable habitat area increases by 4.6% to 5.2% by the 2050s and 2090s compared to the current distribution (Table 3). The increased area is primarily in moderately suitable habitats, while the reduced area is concentrated in highly suitable habitats. Currently, the moderately suitable habitat area is 35,175 km^2^, and the highly suitable habitat area is 158,475 km^2^. Under the SSP126 scenario in the 2050s, the moderately suitable habitat area expands to 49,625 km^2^, increasing from 6.3% to 9.1%. In contrast, the highly suitable habitat area decreases to 146,350 km^2^, shrinking from 28.5% to 26.9% (Table 3). These findings suggest that, although moderately suitable habitats for *E. cardinalis* expand significantly under future climate scenarios, this expansion is largely driven by the degradation of highly suitable habitats.

## 4. Discussion

### 4.1. Accuracy Accessment of the Model and Present Distribution of E. cardinalis Habitats

*E. cardinalis*, a fish of notable commercial significance, characterized by a large population and high economic worth within the NSCS, is directly influenced by both fishing pressure and various marine environmental factors [29,62]. Despite this, the research exploring the impacts of CC on *E. cardinalis* has been rather scarce to date. In this study, leveraging future climate data along with SDMs, five single models and four ensemble models were constructed. The intention was to simulate the current distribution of *E. cardinalis* and also to forecast the changes that might occur in its potential habitat under future climate scenarios. It turned out that the four ensemble models surpassed the five single models in performance, as manifested by their higher TSS and ROC values. This result is in line with the previous studies conducted on other species [9,47,63], thereby highlighting the remarkable versatility of ensemble models in predicting the distributions of different species. The primary advantage of ensemble models lies in their ability to combine predictions from multiple individual models, thereby improving accuracy [64]. This approach enhances performance by mitigating overfitting, a common issue in single models, particularly when dealing with large datasets or complex features [65,66]. Moreover, ensemble methods reduce the impact of outliers and noise, resulting in more stable and reliable predictions [51]. Despite their widespread application in ecological research, SDMs face several limitations. Lower-resolution environmental data may fail to capture critical ecological details, thereby reducing the prediction accuracy [67]. In contrast, high-resolution data provide richer spatial information, improving the model precision and spatial accuracy [68,69]. However, traditional SDMs primarily focus on static spatial distribution predictions and often overlook dynamic factors such as species’ biological characteristics, ecological processes, and population dynamics [70]. This limitation hampers their ability to predict long-term adaptive changes in species under evolving environmental conditions [71]. To address these challenges, future SDM research should place greater emphasis on modeling interspecies interactions and integrating ecological niche theory and adaptive biology [72]. Such advancements will enhance the ability of SDMs to simulate the habitat selection and survival strategies of species under climate change scenarios. This approach can offer more accurate predictions of species distributions within specific ecosystems and provide more reliable decision support for biodiversity conservation and invasive species management [73,74,75]. Furthermore, incorporating internal ecosystem dynamics, species adaptability, and niche shifts into SDMs will enable better predictions of how species adjust to environmental changes, supporting efforts to preserve biodiversity in the face of climate change [76].

The ensemble model based on the majority voting algorithm predicts that the currently suitable habitats of *E. cardinalis* are widely distributed in the NSCS within water depths of 110 m. Highly suitable habitats are predominantly concentrated in the Beibu Gulf, particularly north of 20° N. These findings align with previous research [62], further demonstrating the ability of ensemble models to accurately simulate the *E. cardinalis* distribution and predict future habitat changes under varying climate scenarios. The current distribution of *E. cardinalis* shows significant spatial variability. The probability of occurrence peaks at a water depth of 44 m and gradually declines as the depth increases. Similarly, Chen reported that *E. cardinalis* in the Beibu Gulf is primarily distributed at depths of 30–60 m, with a maximum depth of 124 m [26], consistent with the findings of this study. In this study, BM, SSS, and SSH were selected as key environmental factors for model construction. Among these, BM is the most significant factor influencing the occurrence of *E. cardinalis*. SSS and SSH, on the other hand, primarily affect changes in its abundance [26,29]. The response curve of SSS reveals two peaks at 32.5‰ and 33.3‰, suggesting that the probability of fish occurrence is highest at these salinities. This may relate to the life history stages of *E. cardinalis*: juvenile fish are densely concentrated in nearshore waters with depths less than 30 m [24], while mixed distributions of juvenile and adult fish occur between 30 and 60 m, and adult fish dominate waters deeper than 60 m [26]. Additionally, SSH impacts the ocean circulation and water mass distribution, promoting upwelling and nutrient convection. These processes positively influence benthic habitats, enhancing growth and reproduction conditions for *E. cardinalis* [40].

### 4.2. The Influence of Future Climate Scenarios on the Habitat of E. cardinalis

The findings of this research suggest that, in the context of future climate conditions, the habitat of *E. cardinalis* demonstrates a propensity to expand towards greater depths and higher latitudes, which is in accordance with the typical migratory patterns of marine organisms in response to CC [77,78]. Among the crucial environmental determinants, alterations in SSH and BM are comparatively minimal under future climate projections [79], thereby rendering SSS the principal factor influencing the distribution of *E. cardinalis*. The absolute salinity within the Pacific Ocean has been on a long-term downward trajectory, which is congruent with the future SSS projections in the study area [80]. Under the SSP370 scenario, the salinity range within the study area is anticipated to diminish from 14.22–33.70‰ in the 2050s to 13.96–33.53‰ in the 2090s. Despite an overall expansion in suitable habitat areas under future climate conditions, highly suitable habitats are expected to decline significantly, a pattern similar to that observed in mackerel [40]. For example, under the SSP126 scenario in the 2090s, the highly suitable habitat area is projected to decrease from the current 158,475 km^2^ to 146,350 km^2^, marking the largest reduction among the scenarios. The expansion of suitable habitats into high-latitude and deep-sea areas may reflect the increasing availability of suitable spawning grounds at higher latitudes and the expanded activity range of adult fish in deeper waters. During spawning, scattered fish gather in nearshore shallow waters to form dense reproductive schools. Juvenile fish typically grow and fatten in coastal shallow waters before gradually migrating to deeper areas as they mature [26].

The impact of CC has driven the expansion of suitable habitats for *E. cardinalis* towards coastal areas, potentially altering ecological niches and profoundly affecting the marine ecosystem in the NSCS [81]. Predators at higher trophic levels may be forced to migrate to new areas or adapt by seeking alternative prey, leading to the formation of new ecological communities [82,83]. As a fluctuating species, *E. cardinalis* has experienced significant population changes over the past 40 years [26,84]. Such fluctuations could influence the sustainability and productivity of fisheries in the NSCS, potentially reducing the region’s future fishery potential [85]. Given its ecological significance, *E. cardinalis* plays a pivotal role in understanding the impact of CC on the fisheries and ecosystem dynamics of the NSCS. The projected changes in the distribution and population of *E. cardinalis* underscore the urgent need for forward-looking and effective management strategies. These measures are essential to ensure the sustainable development of the marine ecosystem, particularly in the face of dynamic changes driven by CC [86].

Population growth and rising wealth levels are driving increased demand for food and resource-intensive diets, underscoring the urgent need for a transformation towards more efficient, inclusive, resilient, and sustainable aquatic food systems [87]. The *Blue Transformation Roadmap* (2021–2030) highlights the importance of aligning aquatic food systems with the Sustainable Development Goals (SDGs). This includes establishing sustainable value chains across social, environmental, and economic dimensions, supporting livelihoods, and ensuring equitable benefit distribution [88]. Globally, fisheries provide employment for over 10% of the coastal population, particularly in tropical regions [89,90]. Marine capture fisheries are a critical source of food and jobs in developing countries [91]. However, women face significant disadvantages within the fishery value chain. According to the Food and Agriculture Organization of the United Nations (FAO), women account for only 21% of employment in the fishery sector, with most working in informal roles, resulting in high opportunity costs and persistent inequities [92,93,94,95]. Current research often prioritizes the utilization of marine resources, such as fish, while overlooking the rights and needs of vulnerable groups [96]. Addressing these inequities requires the establishment of fair and just institutional frameworks that prioritize the perspectives of marginalized groups [97]. An integrated interdisciplinary approach to marine sustainability is gaining recognition as a key strategy [98]. Such an approach can drive collaborative interventions for marine sustainability and governance, ensuring inclusivity and equity in resource management [99].

## 5. Conclusions

This study employs SDMs to assess the potential distribution of suitable habitats for *E. cardinalis* under current and future scenarios in the NSCS. The results demonstrate that ensemble models outperform single models in predictive accuracy. At present, the suitable habitats for *E. cardinalis* are predominantly concentrated in the Beibu Gulf, with BM and SSH identified as the primary factors influencing its distribution. Under future climate scenarios, suitable habitats for *E. cardinalis* are projected to degrade in certain nearshore areas while expanding towards higher latitudes and deeper waters. However, a significant portion of highly suitable habitats is expected to degrade, highlighting potential challenges for the species’ future sustainability. Currently, the model primarily addresses the environmental impacts on *E. cardinalis*, without considering other critical factors such as biological interactions and human-induced drivers. Future research should focus on integrating these factors into SDMs to enhance model accuracy and provide a more comprehensive understanding of the species’ distribution dynamics. Incorporating such variables will improve predictions and support more effective conservation and management strategies.

## Figures and Tables

**Table 1:** Accuracy evaluation of individual species distribution models (SD1 is the standard deviation of ROC; SD2 is the standard deviation of TSS). The meanings represented by the abbreviated letters: flexible discriminant analysis (FDA), generalized additive model (GAM), generalized linear model (GLM), multivariate adaptive regression splines (MARS), maximum entropy network, receiver operating characteristic (ROC), true skill statistic (TSS), standard deviation (SD).

Evaluation	FDA	GAM	GLM	MARS	MAXNET
ROC	0.957	0.954	0.898	0.967	0.942
SD1	0.019	0.020	0.029	0.021	0.014
TSS	0.846	0.822	0.720	0.860	0.783
SD2	0.052	0.073	0.053	0.060	0.038

**Table 2:** Accuracy assessment of four ensemble models. ROC and TSS are indices for evaluating models, with their values ranging from 0 to 1. The larger the ROC and TSS values, the better the performance of the model.

Assessment	EMca	EMmean	EMmedian	EMwmean
ROC	0.97	0.962	0.961	0.962
TSS	0.85	0.85	0.833	0.85

**Table 3:** The area of different suitable habitats for *E. cardinalis* in the NSCS (km^2^).

Period and Climate Scenarios	No Suitable Areas (0 < HSI ≤ 0.25)	Low Suitable Areas (0.25 < HSI ≤ 0.5)	Moderately Suitable Areas (0.5 < HSI ≤ 0.75)	Highly Suitable Areas (0.75 < HSI ≤ 1)
Current	309,225	53,700	35,175	158,475
SSP-126-2050	293,950	54,825	49,625	146,350
SSP-126-2100	293,550	55,025	47,050	149,125
SSP-370-2050	294,100	53,575	49,325	147,750
SSP-370-2100	294,700	53,275	45,975	150,800
SSP-585-2050	294,475	52,650	48,400	149,225
SSP-585-2100	293,325	54,400	49,175	147,850

## Data Availability

The dataset is available on request from the authors.
